# Can Morning Rise in Salivary Cortisol Be a Biological Parameter in an Occupational Rehabilitation Clinic? A Feasibility Study

**DOI:** 10.1155/2014/793641

**Published:** 2014-03-05

**Authors:** Kari Storetvedt, Anne Helene Garde

**Affiliations:** ^1^The Occupational Rehabilitation Centre in Rauland (AiR), 3864 Rauland, Norway; ^2^Department for Physical and Rehabilitation Medicine, University Hospital of Northern Norway (UNN), 9038 Tromsø, Norway; ^3^National Research Center for the Working Environment, Lersø Parkalle 105, 2100 København Ø, Denmark

## Abstract

*Objective.* To test the feasibility of measuring salivary cortisol in an inpatient clinic for occupational rehabilitation, and cortisol as a biological parameter. *Methods.* In 17 patients in vocational rehabilitation, cortisol in saliva was measured at awakening, 30 min after and before bedtime. The cortisol measures were taken on day 2 and day 22 of the rehabilitation period. Cortisol awakening response was estimated in absolute value and as percent rise of the value at awakening. *Results.* The cortisol awakening response in absolute value was 6.7 (SD = 4.9) nmol/L on day 2 and 2.7 (SD = 5.6) nmol/L on day 22. The change was not statistically significant. The mean value for cortisol morning rise calculated in percent was 186% on day 2 and 51% on day 22. *Conclusion.* It is possible to conduct a clinical study including salivary cortisol in a rehabilitation clinic. This study indicates that cortisol morning rise may be a useful biological parameter for effect of intervention in a rehabilitation clinic; this remains to be tested in a larger population.

## 1. Introduction

Patients in occupational rehabilitation clinics often present with long lasting musculoskeletal pain and depression, with perceived job stress and general life stress. Musculoskeletal pain and psychiatric diagnosis are among the most frequent causes for sick leave and disability pension in Norway; see Brage et al. [1] and Knudsen et al. [2]. Through the last forty years the role of cortisol in stress regulation and as a potential factor in development of chronic pain and stress related disease has been explored but is still sparsely understood. The pattern of cortisol activity has been shown to vary with both psychological and biological factors in several studies; some of them are referred below. The possibility to measure cortisol in saliva has given new options for using cortisol as a biological parameter in clinical studies.

The rise in free cortisol measured in saliva from the time of awakening and to the peak value or through the first hour after awakening has been termed the cortisol awakening response (CAR). The CAR has been found useful as a measure of reactivity of the hypothalamic-pituitary-adrenal (HPA) axis as described by Pruessner et al. [3] and Wilhelm et al. [4].

In their literature review of saliva cortisol studies, M. Kristenson et al. [5] address both the great interest in using saliva cortisol measurement in research on health, disease, work stress, and social differences and also the frustrations in opposing and ambiguous results.

Several studies have addressed the effect of work related stressors on the CAR. Experienced chronic stress, self-perceived work overload, depressive symptoms, and worrying were associated with higher CAR in studies by Pruessner et al. [6] and Schlotz et al. [7]. Morning cortisol has been related to recovery from work in a study by Gustafsson et al. [8], and Harris et al. [9] found CAR related to reestablishing of normal diurnal rhythm after shift work.

In a meta-analysis, Chida and Steptoe [10] found that CAR was positively associated with job stress and general life stress but negatively associated with fatigue, burnout, and exhaustion. In major depression (MDD) Hsiao et al. [11] found that a higher level of self-reported depression correlated with lower cortisol at awakening time and higher evening value. In fibromyalgia low cortisol on awakening was found by Riva et al. [12]. Cortisol concentration in the first hour after awakening was inversely related with level of posttraumatic stress disorder (PTSD) symptoms in a study by Neylan et al. [13], and Roberts et al. [14] found a lower cortisol during 60 min postawakening, in 56 patients diagnosed with chronic fatigue syndrome (CFS).

A vulnerability to chronic muscle pain could have a background in genes involved in adrenergic control and reaction to stress, as shown by Skouen et al. [15]. A recent study from Canada by Vachon-Presseau et al. [16], including 16 patients, found that patients with chronic back pain had higher levels of cortisol on five measures during the day, including time of awakening and 30 min thereafter.

A prospective study from England by McBeth et al. [17] showed that high cortisol after Dexamethasone 0,25 mg could predict development of chronic widespread pain (CWP). This was also true for low cortisol in the morning and high cortisol in the evening, indicating a role of cortisol regulation in development of chronic widespread pain. Geiss et al. [18] looked at cortisol in the morning in a group of patients suffering from persistent sciatic pain 8 weeks after discectomy. They found a lower cortisol immediately after awakening but a higher rise 30 min thereafter (CAR) compared with control group. The high CAR was associated with elevated interleukin 6 (IL-6). There is a close relationship between the stress system components regulated by the HPA-axis, the sympathetic nervous system (SNS), and the immune system as described by Chrousos [19], Glaser and Kiecolt-Glaser [20], and Segerstrom and Miller [21].

Pathophysiology and psychological factors behind chronic widespread pain, leading to disability are items of great importance. Such conditions lead to individual suffering and society costs; see Brage et al. [1] and Knudsen et al. [2]. Searching for interventions that could reduce work disability risk will be of great importance. There is a great need for studies aimed at better understanding of the physiology and psychological factors behind chronic pain conditions. Likewise a biological measurable parameter indicating effect of an intervention could be of great interest to the rehabilitation field. Could cortisol, with its widespread and complex biological influence be such a measurable biological parameter?

This feasibility study aimed to see if it would be possible to use salivary cortisol measures in a 24-hour-based occupational rehabilitation clinic, in a distant rural region.

These questions were asked: would patients in this occupational rehabilitation clinic, with different diagnoses and often with overlapping symptoms, as measured with the Subjective Health Complaint (SHC) inventory, described by Eriksen et al. [22], have disturbed cortisol diurnal rhythm at a group level? And if so, could any change in cortisol rythm be detected after the twenty days rehabilitation periode (time between cortisol measures)?

In spite of a small number of participants, the authors and coworkers would also look for correlations between cortisol measures and health complaints in the SHC, perceived memory and attention, sleep parameters, duration of sick leave, number of pain sites, and body mass index (BMI).

## 2. Method

### 2.1. Participants

A total of 17 patients (51% of the patients invited) at the rehabilitation clinic, 12 women and 5 men, participated in the study. The mean age was 45, ranging from 32 to 60 years. Two groups of patients who arrived in summer and early autumn in 2012 were invited to participate. One patient had ischias with discectomy, four had only psychiatric diagnoses, and 12 had combined somatic and psychiatric diagnoses, including one with CFS and one with fibromyalgia. The most common psychiatric diagnosis was depression (*n* = 11/17), mild (*n* = 9) or moderate (*n* = 2). Informed consent was obtained from all participants prior to taking part in the study. All data were obtained according to the Helsinki Declaration. The Medical Ethics Committee, Region South-East in Norway, approved this study.

### 2.2. Procedure

Saliva cortisol rhythm was measured on days 2 and day 22, of a 4-week rehabilitation program in a patient group with compound health problems and health complaints. Test days 2 and 22 were chosen for practical reasons and were days without a very challenging program. Patients were invited to participate on the first day of arrival to the clinic. Those who agreed to participate were given a questionnaire together with 6 small plastic tubes (without a swab, delivered to the clinic from our collaborating laboratory) for salivary sampling. Oral and written information was given, about how to collect saliva samples for cortisol analyses. Questionnaires were handed in to the first author along with the first three test tubes on the third day at the clinic. Diagnosis at arrival, diagnosis given at end of the stay, duration of sick leave, and medication used during the stay were obtained from the patient journal at the clinic.

### 2.3. Saliva Cortisol Sampling

Sampling of cortisol was done on a Friday, the second day after arrival, and on the twenty-second day, a Thursday, 6 days before leaving the clinic. Patients were instructed to refrain from eating, drinking, smoking, and tooth brushing the last 30 min before sampling. The three samples were collected immediately after waking up in the morning (I), 30 minutes thereafter (II), and before going to sleep at night (III). The exact time for sampling was written on a schema and on the labeled test tube. The tubes with saliva were kept in the participant's room until next morning. Then samples were gathered and placed in a freezer (−20°C) for one or four weeks, or in a refrigerator for two or three days, before they were transported to the laboratory under cooling conditions. Salivary cortisol has been shown by Garde and Hansen [23] to have great stability under storing conditions.

### 2.4. Questionnaire

Background variables, including gender, age, BMI, smoking and menstrual data for women, and presence of hypertension, hypothyreosis, airway allergy, food intolerance, and frequent infections were registered from questionnaires.

Health complaints were measured with the subjective health complaints (SHC) inventory that cover 29 questions concerning common health complaints experienced during the last 30 days; see Eriksen et al. [22]. In addition to the SHC questionnaire we asked for perceived cognitive function as problem with attention and memory and about pain in hands, hips, knees, legs, or feet during the last months. All items were answered on a four-point scale (1: not at all, 2: a little, 3: some, and 4: severe). We counted pain sites according to the study of Kamaleri et al. [24], where a number of pain sites were correlated with later disability pension, in a strong way. On the same day when participants collected saliva samples, they registered wakeup time, exact time for collecting saliva, the last nights total sleep time, numbers of wakeups during the night and time before falling asleep.

### 2.5. Multidisciplinary Occupational Rehabilitation Program

This study was conducted at a national occupational rehabilitation centre offering a four-week inpatient multidisciplinary rehabilitation program. The rehabilitation team is made up of physician, nurse, physiotherapist, vocational social worker, and sports educator. The program is tailored to improve the patients' level of functioning and work ability with an emphasis on physical activity, counseling psychology, mindfulness, and cognitive behavioral modifications. The main aim is to improve the person's self-efficacy, self-esteem, and coping and resilience related to family and working life along with strengthening physical fitness. Balancing physical training with rest and restitution is an important theme.

### 2.6. Cortisol Analyses

After arrival in the laboratory, all saliva samples were stored at −20°C until analysed. At the day of analysis, the samples were left to thaw at room temperature for approximately 45 min and centrifuged at 3500 g for 10 min. Liquid-liquid extraction of 200 *μ*L saliva with 1 mL ethyl acetate, evaporated to dryness under nitrogen flow and redissolved in 200 *μ*L 10% methanol (MeOH), was carried out as described by Jensen et al. [25]. D-4-cortisol was used as internal standard. The calibration range was 0.5–90.0 nmol/L.

### 2.7. Determination of Cortisol

A volume of 25 *μ*L was injected into an Agilent 1200 HPLC (Agilent technologies, Santa Clara, CA, USA) equipped with a C18 2.1 × 50 mm 2.6 *μ*m Kinetex column and a Krud-katcher ultrafilter (Phenomenex, Torrance, CA). The mobile phase consisted of a 2 mM aquatic solution of ammonium acetate with 0.1% (v/v) formic acid (A) and MeOH with 2 mM ammonium acetate and 0.1% (v/v) formic acid (B). A linear gradient was run over 3 min from 10% to 100% B and maintained at 100% MeOH for 1.5 min, followed by 2 min of equilibration at 10% MeOH resulting in a total run time of 6.5 min. The flow rate was 0.5 mL/min and the temperatures of the autosampler and column oven were 8°C and 40°C, respectively. Detection of cortisol was performed by a mass spectrometer: an Agilent 6460 QQQ (Agilent technologies, Santa Clara, CA) equipped with a jet stream ESI ion source was operated in the positive ion mode as described by Jensen et al. [25]. The transitions were *m*/*z* 363.2→*m*/*z* 121.1 for cortisol; *m*/*z* 367.2→*m*/*z* 121.2 for D-4-cortisol.

To show equivalence between different runs, natural saliva samples (1.96 nmol/L and 8.08 nmol/L) were used as control materials and analyzed together with the samples. Westgard control charts (see Westgard et al. [26]) were used to document that the analytical method remained under analytical and statistical control—in other words, that the trueness and the precision of the analytical methods remained stable.

### 2.8. Statistical Procedures and Data Analysis

Absolute cortisol values in nmol/L were used in statistical analyses, without a logarithmic transformation.

CAR was estimated as the difference between the cortisol value measured 30 minutes after awakening and the value immediately after wakeup, in absolute value (CARi).

At a group level CAR% was defined as the mean value of CARi in percent of the mean cortisol value at wakeup and estimated from the mean cortisol values, according to Pruessner et al. [3] and Wust et al. [27]. The cortisol slope during the day was estimated in two different ways; the low slope defined as the difference between the cortisol value on awakening and the evening sample, and the high slope, defined as the difference between 30 minutes after wakeup and the evening sample. SPSS (version 20) was used for statistical analyses. Differences in cortisol levels before and after the rehabilitation program were analyzed using paired sample *t*-test. Correlations between health-related factors, attention, memory, pain and cortisol variables at baseline were investigated using Pearson product—moment correlation analyses.

## 3. Results

### 3.1. Background Variables

The mean BMI was 30 kg/m^2^ with a range from 21 kg/m^2^ to 41 kg/m^2^. Less than 20% reported to be smokers (19%), to have hypertension (12%), hypothyreosis (12%), food intolerance (12%), airway allergy (18%) or frequent infections (18%).

### 3.2. Saliva Cortisol Measures

All 17 participants delivered the 6 saliva samples as planned. Mean time of awakening was 06.30 (range 04.25–07.30) in the start, and 06.55 (range 05.30–07.40) in the end of the stay. The mean CARi on day 2 was 6.7 nmol/L (sd = 4.9) and on day 22 mean CARi was 2.7 nmol/L (sd = 5.6) (Table 1). This difference or other differences found between cortisol variables in the beginning and at the end of the rehabilitation period, were not statistic significant.

**Figure 1:**
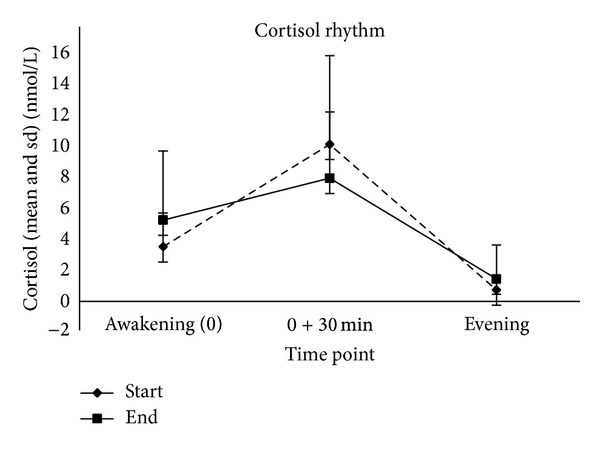
The figure shows that the mean rise in cortisol in the morning (CARi) changed from 6.71 nmol/L (sd = 4.86) in the start of the intervention to 2.74 nmol/L (sd = 5.61) at the end of the stay, and the mean rise in percent of the value at awakening (CAR%) changed from 186% in the beginning to 51% in the end of the stay.

The mean calculated CAR% changed from 186% at the start to 51% at the end of the stay (Figure 1).

### 3.3. Recording of Symptom Load/Questionnaires

On the subjective health complaints (SCH) inventory, almost all (94%) reported musculoskeletal complaints and 76% reported gastrointestinal complaints, during the last 30 days. In the SHC category called pseudoneurology, this patient group also have high scores, 90% with tiredness, 75% with depression and 65% with sleep problems. This patient group shows, according to SHC answers, a great symptom load compared to the general population in Norway. (The SHC inventory was filled in only once, in the beginning of the stay. This questionnaire, measuring complaints during the last 30 days, is not suitable for repeated measures in the beginning and end of a three week period). Perceived cognitive problems were common, with attention problems in 90% and problem with memory in 75%.

Pain sites were counted according to Kamaleri et al. [24]. Of the ten pain sites chosen, head, neck, shoulders, arms, hands, upper back, lower back, hips, knees, and feet, 10 patients (59%) reported 5–10 pain sites and 7 patients (41%) reported 0–4 pain sites.

There were no statistical significant correlations between symptoms in SHC, number of pain sites, attention, memory, or time on sick leave, and cortisol variables as measured in this study group. Neither recorded time of awakening or other sleep variables were correlated with cortisol values in this study.

## 4. Discussion

This study was carried out as a pilot study in a clinic for occupational rehabilitation with a small number (*n* = 17) of subjects participating. The study was accomplished without unforeseen problems and with little personal resources required.

Two days after arrival to the clinic, the mean rise in cortisol following awakening (CAR) in percent of the value at wakeup was high, 186% (Figure 1). Pruessner et al. [3] found in their studies of 152 subjects that cortisol increased by 50–75% within the first 30 minutes after awakening. Wust et al. [27] confirm this result in their study of 509 subjects, finding a mean cortisol increase of about 50% within the first 30 minutes. They mention a great interindividual variation in CAR.

In their review Chida and Steptoe [10] found that CAR was positively correlated with job stress and general life stress. This could explain the high CAR on arrival in our patient group, attending vocational rehabilitation due to problems coping with work, health problems, and general life problems. A high CAR has been found in persistent sciatic pain by Geiss et al. [18], while a group with cronic back pain studied by Vachon-Presseau et al. [16] showed high cortisol as well at wake up as after 30 min. In this actual study 65% reported lower back pain the last month. This could possibly contribute to the high CAR in our patient group.

A possible confounder for high cortisol values at arrival could be an activation of HPA axis with high CAR, as described by Kirschbaum and Hellhammer [28] and Kudielka et al. [29], due to the actual situation, including newly arrival at the clinic, new environments with expectations, and anxiety about the rehabilitation program and also included in this study.

Chida and Steptoe [10] found that CAR is negatively related to fatigue, burnout or exhaustion. In syndromes as fibromyalgia, CFS and PTSD Riva et al. [12], Roberts et al. [14] and Neylan et al. [13] found low cortisol response to awakening. Possibly such an expected negative influence on the CAR in this patient group could be overshadowed by a great patient number with a high CAR.

Results two days after admission also show a low cortisol value at wakeup (Figure 1). Low cortisol at wakeup has been found in patients with persistent sciatic pain after discectomy by Geiss et al. [18], in fibromyalgia by Riva et al. [12], and also in primary insomnia by Backhaus et al. [30]. Pain conditions and sleep problems could possibly contribute to the low cortisol value at awakening in the actual patient group two days after admission.

As a confounder, the time or hour of awakening could have an impact on the cortisol values measured at wakeup. Patients on sick leave recently admitted to the clinic could be used to a later wakeup time at home and after adapting to the clinics schedule they maybe wake up on an earlier stage of the natural rising cortisol curve during the late night, as described by Williams et al. [31].

Throughout the last decade many authors have strained the high grade of comorbidity and overlap of symptoms in patients suffering from symptom based conditions as chronic muscelosceletal pain, fibromyalgia, other chronic pain, depressive disorder, post traumatic stress disorder and irritable bowle syndrome, see Kato et al. [32], Cohen et al. [33], and Croft et al. [34]. Such overlap of symptoms was confirmed in this patient group from occupational rehabilitation, with high symptom score on the SHC inventory and supplying questionnaire.

It has been proposed that these sparsely understood conditions with different diagnoses can have common causes and common pathophysiologic pathways. Such mechanisms can according to Getz et al. [35] involve as well biological as cognitive and emotional factors. Kirkengen and Ulvestad [36] discuss if the reason why the pathophysiological mechanisms and disease processes in such conditions are still sparsely understood could be a lack in coherence with “the biomedical paradigm of understanding”. Such thoughts resulted in the idea to study these patients at a group level and not divided due to different diagnoses.

Long lasting stress load can influence the immune system. Cortisol is assumed to take part in regulation of immune processes and can be a link between stress and the immune system; see Segerstrom and Miller [21] and Glaser and Kiecolt-Glaser [20]. This can possibly be a pathway for disease in pain conditions, with low grade of inflammation in muscle, tendons, and joints. Cortisol can have an inhibiting as well as an activating effect on inflammation. High cortisol due to long lasting stress can activate inflammation processes. This effect exists in peripheral tissues but especially in the central nervous system (CNS). Cortisol can influence cognitive processes in the hippocampal area and also inhibit glucose entrance to nerve cells according to Sorrells and Sapolsky [37]. These mechanisms can possibly be of importance to cognitive problems, as problem with attention and memory, often experienced by patients in the occupational rehabilitation clinic.

Chrousos [19] suggests that symptoms in pain and fatigue syndromes (as fibromyalgia and CFS) could be due to unbalance between the immune reactions and the stress response, resulting in a “sickness syndrome” with fatigue, depression, social withdrawal, hyperalgesia, and “sickness behavior”.

In this pilot study, the mean diurnal cortisol curve in the end of the rehabilitation program, on day 22, shows a CAR close to previously reported normal value for CAR, 51% rise in cortisol in the first 30 minutes, as described by Pruessner et al. [3] and Wust et al. [27]. In contrast, a 186% rise in cortisol (CAR) was observed on the second day of the stay (Figure 1), which indicates a more dysregulated HPA axis function. Although not statistically significant it may be speculated that this change in the morning CAR could be related to the intervention. The lack of statistical significance could be due to the small number of participants in this pilot study, and the results could come out differently in a larger patient group.

It remains to be further explored in an extended study with a greater number of patients if a high CAR in a patient group like this is common in the start of rehabilitation, possibly due to high stress load and if a more normal value will be found after the rehabilitation program. If significant results are found, it could indicate an effect of the rehabilitation intervention on HPA axis regulation, cortisol diurnal curve, and morning rise in cortisol (CAR).

An extended study could possibly contribute to a better understanding of the pathophysiology of complex and stress related disease as here addressed. In an extended study with a greater number of patients, it would be of importance to supply the study with measure of the diurnal cortisol rhythm some days or a week before arrival to the clinic, to avoid the possible confounding effect of HPA axis activation shortly after arrival. A control group with cortisol measures a few days before and after a 4-week vacation could be of interest, to compare cortisol rhythm in the patient group, with a group from the working population, and to compare effect of the rehabilitation program with a possible effect of ordinary vacation.

This study shows that it is fully possible to conduct a clinical study including cortisol measures in a rehabilitation clinic, even in a distant area. If change in cortisol morning rise (CAR) may be a useful parameter for effect of intervention in a rehabilitation clinic, remains to be tested in a larger population.

## Figures and Tables

**Table 1:** Saliva cortisol nmol/L (mean value and standard deviation) in the beginning and end of a rehabilitation program.

	Start	End	Sig.
At awakening	3.58 (2.21)	5.33 (4.53)	0.07
30 minutes after wakeup	10.29 (5.81)	8.07 (4.35)	0.25
Evening	0.75 (.48)	1.46 (2.23)	0.19
CARi	6.71 (4.86)	2.74 (5.61)	0.09
Slope high	9.54 (5.51)	6.60 (5.58)	0.18
Slope low	2.84 (2.11)	3.86 (4.65)	0.32

CARi: cortisol awakening response, increase from awakening time to 30 min after.
